# Detection of Staphylococcal Enterotoxins A and E and Methicillin Resistance in *Staphylococcus aureus* Strains From Moroccan Broiler Chicken Meat

**DOI:** 10.1155/2024/2790180

**Published:** 2024-08-26

**Authors:** Sabrine Nacer, Saâdia Nassik, Fatima Zahra El Ftouhy, Sophia Derqaoui, Mohamed Mouahid, Mustapha Lkhider

**Affiliations:** ^1^ Laboratory of Virology Oncology Biosciences Environment and New Energies Faculty of Science and Technology Mohammedia University Hassan II, Casablanca, Morocco; ^2^ Avian Pathology Unit Department of Veterinary Pathology and Public Health Hassan II Agronomic and Veterinary Institute, Rabat, Morocco; ^3^ Laboratory of Biochemistry Environment and Agri-Food Faculty of Science and Technology Mohammedia University Hassan II, Casablanca, Morocco; ^4^ Mouahid's Veterinary Clinic, Temara 12000, Morocco

**Keywords:** broiler chicken meat, MRSA, sea, see, *Staphylococcus aureus*

## Abstract

Foodborne epidemics have become a serious public health emergency worldwide. Foods of animal origin, in particular chicken meat, are considered to be potential vectors of pathogenic bacteria, particularly *Staphylococcus aureus*. This bacterium can be resistant in the form of methicillin-resistant *S. aureus* (MRSA) or produce enterotoxins leading to food poisoning when ingested. This study is aimed at exploring the virulence genes in *S. aureus* responsible for producing enterotoxins (staphylococcal enterotoxin [SE] A [sea] and SE E [see]) and determining the prevalence of MRSA in raw broiler meat in the Casa-Rabat region in Morocco. A quantitative (q) PCR (qPCR) assay, using specific primers for *S. aureus* (nuc) confirmation and detection of enterotoxin genes (sea and see), as well as the methicillin-resistant gene (mecA), was employed. Our findings indicated that all tested strains were positively identified as *S. aureus*. Among them, one isolate (1/54) tested positive for the see gene (1.85%), while none carried the sea gene. Furthermore, the mecA gene, indicative of MRSA, was present in 12/54 of the isolates (22.22%). The potential presence of MRSA in Moroccan poultry meat underscores a public health risk. Thus, stringent measures are imperative to curtail the contamination and proliferation of this bacterium during the slaughtering process, underscoring the importance of continuing research into the prevalence of MRSA colonization among poultry slaughterhouse personnel.

## 1. Introduction

*Staphylococcus aureus* (*S. aureus*), an opportunistic and notorious zoonotic pathogen, is responsible for worldwide outbreaks of food poisoning [1, 2]. It possesses two aggravating characteristics: toxin production and antimicrobial resistance [3]. The consumption of *S. aureus*-contaminated foods, mainly chicken meat, remains the major factor in the development of staphylococcal food poisoning in humans [2, 4]. This contamination arises from poor hygiene during slaughter handling and quality of water used for processing [5]. Therefore, meats and meat products can act as transmission vectors in the transmission of methicillin-resistant *S. aureus* (MRSA) to both butchers and consumers [6].

The treatment of infections caused by *S. aureus* has been further complicated by antimicrobial resistance in bacteria, particularly MRSA [7]. As a result, *S. aureus* has been listed by the World Health Organization (WHO) as one of the “priority pathogens” posing a threat to public health [8]. This threat is compounded by MRSA attributed mainly to the presence of the mecA gene, located on one of staphylococcal cassette chromosome mec (SCCmec), which encodes for penicillin-binding protein 2a (PBP2a) and has a low affinity for most beta-lactam antimicrobials, complicating the treatment of infections [3]. Consequently, the importance of monitoring MRSA in retail meat has been highlighted [9].

In addition, staphylococcal enterotoxins (SEs), including SEs A and E (sea and see), produced by enterotoxigenic strains of *S. aureus*, constitute a superfamily of pyrogenic exotoxins that share structural and functional similarities [10]. In fact, as of today, at least 28 SEs and SE-like toxins have been identified [11], which are of paramount importance and danger. sea, for example, is responsible for the symptoms associated with outbreaks of staphylococcal food poisoning [12, 13] and also contributes to the development of organisms' resistance to heat treatment [14], while the presence of SE E (see) has been implicated in many cases of foodborne illness [12].

The disease is characterized by a rapid onset of symptoms, including nausea, violent vomiting, abdominal cramps, and diarrhea which typically last from 24 to 48 h. Complete recovery usually occurs within 1–3 days, and the illness is usually self-limiting; occasionally, it is severe enough to require hospitalization.

Today, the phenomena of food poisoning, foodborne antimicrobial resistance, and resistance gene transfer represent a growing biological risk, necessitating in-depth research into these issues and their underlying nature.

Furthermore, in Morocco, 146 cases of collective food poisoning (CFP) were reported in 2021, without specifying the agent or foodstuff responsible [15]. Therefore, we have no data on CFP caused by *S. aureus*, in particular MRSA and enterotoxin-encoding genes. This study is aimed at exploring the *S. aureus* virulence genes responsible for enterotoxin production (sea and see) and determining the prevalence of MRSA in broiler meat in the region.

## 2. Material and Methods

### 2.1. Samples and Bacteriological Identification

In this study, we are extending our previous research, “Prevalence and antibiotic resistance of *Salmonella* spp. and *Staphylococcus aureus* isolated from broiler meat in modern and traditional slaughterhouses in Morocco” [16], using PCR methods to further investigate the presence of *S. aureus* and MRSA and also to examine the expression of specific staphylococcal toxin genes (see, sea), to provide valuable information on the pathogenicity of this bacterium.

A total of 54 *S. aureus* isolates were identified in the current study using the microbiological test. The isolates originated from 540 broiler chicken meat samples collected from both traditional and modern poultry slaughterhouses in Morocco as described earlier [16]. The strains were phenotypically confirmed as *S. aureus* based on ISO 6888-1: 1999 standard. For that purpose, 25 g of each neck skin, breast, and thigh sample was placed in a sterile bag containing 225 mL of water peptone buffer and then homogenized using the Stomacher to achieve a 10% stock suspension. Serial dilutions up to 10^−5^ were carried out from the 10^−1^ stock solution.

The prepared Petri dishes were inoculated with 0.1 mL of different dilutions with a sterile glass rake in Baird Parker's selective medium (BK055HA Biokar Diagnostics, Zac de Ther, France) with egg yolk and potassium tellurite (3554205Bio-Rad Marnes-la-Coquette, France) and incubated at 37°C for 24–48 h. Suspected *S. aureus* colonies were then confirmed using the catalase (1840, SOLVAPUR, SOLVACHIM, Morocco) and coagulase tests (6BR0020, Biokar Diagnostics, Zac de Ther, France). Finally, each *S. aureus* species was stored at −80°C in TSB with 20% glycerol, until further molecular analysis.

### 2.2. DNA Extraction

Bacterial DNA was extracted from the samples using a commercial column-based kit Invitrogen™ PureLink™ Microbiome DNA Purification Kit (Life Technologies, Thermo Fisher Scientific, Foster City, CA, USA), following the manufacturer's user guide. Briefly, up to 2 × 10^9^ Gram-positive cells were collected by centrifugation, resuspended in 180 *μ*L of lysozyme digestion buffer containing lysozyme, and thoroughly mixed by brief vortexing. Samples were incubated at 37°C for 30 min; then, 20 *μ*L of proteinase K was added, and 200 *μ*L of PureLink™ genomic lysis/binding buffer and carefully vortexed and incubated at 55°C. After 30 min, 200 *μ*L of 96%–100% ethanol was poured into the lysates. The resulting 640 *μ*L of lysate solution, prepared for swabs, was then placed in collection tubes and centrifuged at 10 000 rpm for 1 min at room temperature. These collection tubes were then replaced, and two rounds of washing were then carried out using the washing solutions supplied with the kit. Finally, the DNA was recovered after the elution step and stored at −20°C until use.

### 2.3. Quantitative (q) PCR (qPCR) Assay

qPCR was employed to detect and quantify the nuc, mecA, see, and sea genes in broiler chicken meat samples, as described in previous studies [17–19], and to verify the efficiency of the qPCR reaction, we used a positive control (reagents and known target DNA). For that, each qPCR reaction was set up in duplicate, with each well containing 10 *μ*L of the reaction mixture, comprising 2.2 *μ*L of the test sample and 7.8 *μ*L of the one-step mix. This mix included nuclease-free water, specific forward and reverse primers for each target gene [20–22] (Table 1), and SYBR Green (Applied Biosystems, Life Technologies, Burlington, ON, Canada).

After sealing the plate with adhesive film, it was placed in a thermal cycler (Agilent AriaMx Real-Time PCR System) according to the melting temperature of the primers for all the genes (Table 2). The values obtained are expressed as quantification of qPCR cycle numbers (Cq). Unlike established “Cq cut-off” values for pathogens such as *Salmonella* and *Campylobacter*, any sample registering a Cq value greater than 35 in this study was deemed negative.

## 3. Results

### 3.1. *S. aureus* Confirmation

**Figure 1:**
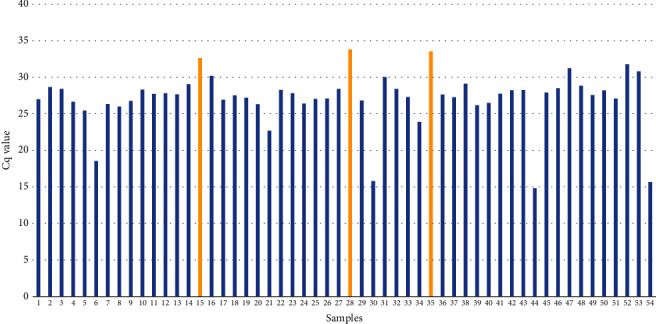
Presence of *Staphylococcus aureus* with corresponding Cq value in broiler chicken meat.

All 54 samples, initially identified as *S. aureus* positive using the microbiological method, were subsequently confirmed as *S. aureus* (100%) using the qPCR method. The Cq values for these samples ranged from 14.80 to 33.81 (Table 3 and Figure 1).

### 3.2. SE (sea, see) Screening

**Figure 2:**
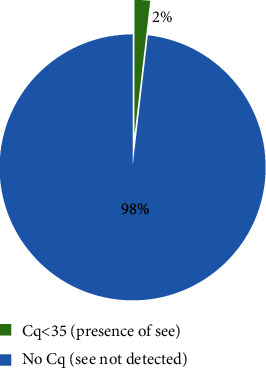
Presence of staphylococcal enterotoxin E (see) in broiler chicken meat.

Among the isolates of *S. aureus* positive for SE genes (sea, see), one isolate (1/54) was positive to see (1.85%), although none of these *S. aureus* colonies carried sea gene (Table 4 and Figure 2).

### 3.3. MRSA Prevalence from Broiler Chicken Meat

**Figure 3:**
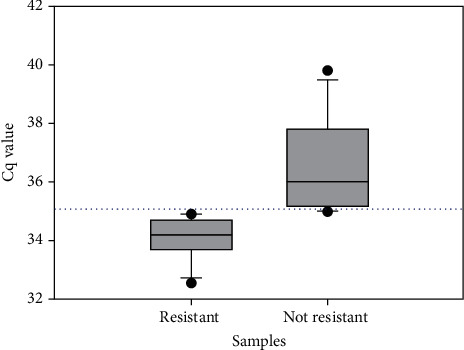
Presence of MRSA with corresponding Cq values in broiler chicken meat.

Overall, out of 54 positive *S. aureus* samples, 12 samples (22.22%) were resistant to methicillin, with a Cq value ranging from 32.59 to 34.94. Also, 17 samples (31.48%) showed no resistance with a Cq value ranging from 35 to 39.42. (Table 5 and Figure 3).

## 4. Discussion

*S. aureus* contamination in food, particularly in chicken meat, results from inadequate hygienic handling and processing. It constitutes a potential risk to public health due to its capability to produce enterotoxins [3, 24], which are major virulence factors of *S. aureus*, particularly concerning food safety [25]. On the other hand, the fact that food, intended for consumption, may contain MRSA is a real disaster, as MRSA strains are considered to be “super-bacteria” in the health field [26]. In Morocco, there are no consistent data concerning MRSA and the virulence genes see and sea in broiler chicken meat.

In the present work, all 54 broiler meat samples that tested positive for *S. aureus* using the microbiological method were also confirmed by qPCR, attributed to the presence of the nuc gene amplicon. Our findings indicated that 1.85% of the isolates were enterotoxigenic, containing only one gene. This contrasts with studies from Turkey [27] and China [28], where 69% and 46% of *S. aureus* isolates from raw chicken meat tested positive for one or more toxin genes, respectively.

Of the two virulence genes examined (sea and see), the see gene was detected, with a prevalence of 1.85% (1/54). This rate is lower than those reported in Nigeria [29] and Kaliobia Governorate [30]. Interestingly, studies from Chennai, India [5], and Thailand [9] align with our findings, reporting no *S. aureus* isolates carrying the sea gene, whereas another study conducted on frozen chicken meat revealed that three out of six samples (50%) were enterotoxigenic, while two strains produced sea [31].

The variation found in the predominant enterotoxin genes of *S. aureus* in these different studies might be influenced by geographical differences, which could be further affected by the different ecological origins of the isolated strains [28, 32].

Since the first characterization of sea in 1959, five SEs, named sea to see, have also been identified on the basis of differences in SE antigenicity [13, 33, 34]. These SEs represent emetic toxins and are one of the causes of food poisoning in humans [35]. SEs have been classified as members of the superantigen family of pyrogenic toxins because of their biological activities and structural relatedness [36].

Indeed, it is known that about 95% of staphylococcal food poisoning outbreaks are caused by SE types sea to see [34]; they are responsible for the clinical manifestations of staphylococcal food poisoning and a septic shock-like illness, and their ingestion leads to severe gastroenteritis with emesis, nausea, and diarrhea [37].

On the other hand, the evolution of *S. aureus* in the antibiotic era has revealed the emergence of virulent strains, many of which have acquired resistance to methicillin, representing a real threat to human health [38]. Our findings show that MRSA was detected in 22.22% of broiler meat samples from slaughterhouses. Several studies carried out in different parts of the world and concordant with us have recorded higher prevalence rates: 29.9% in Nigeria [29], 35.4% in the Czech Republic [39], 38% in Egypt [7], and 56% in South Africa [40]. Lower prevalence rates have also been reported: 1.3% in Canada [41], 4.3% in South Africa [42], 1.7% in China [43], and 0.3% in Korea [44]. This difference might be linked to the different slaughtering conditions or the strategies used to combat antibiotic resistance.

It should also be noted that a previous study carried out on live poultry in Northern Morocco did not reveal the presence of MRSA [38].

In recent years, antibiotic-resistant bacteria have received considerable attention due to their immediate risk to public health, and MRSA is no exception. The presence of this bacterium in poultry and poultry products not only increases the incidence of foodborne outbreaks but also represents a risk of horizontal transmission of resistance between bacterial strains, as this means that even strains that have never been exposed to antibiotics can acquire this resistance through gene transfer [45], rather than through the selection pressure exerted by the excessive use of antibiotics.

The burden of foodborne disease caused by MRSA and SEs is likely to continue to increase in the coming years. Although one of the common superantigen genes, sea, was not detected in our study, there are probably other types of superantigen than see. In addition, these results underline the presence of pathogenic MRSA in Moroccan broiler chicken meat. This emphasizes the imperative need to implement robust preventive measures to control the spread of methicillin-resistant strains, as well as to minimize the presence of potentially dangerous enterotoxin-producing staphylococci. These constatations also highlight the need to improve hygiene practices throughout the food production chain, from farming to consumption, in order to reduce the risks to public health.

## 5. Conclusion

To the best of our knowledge, this study is the first to report the presence of the enterotoxin gene (see) at 1.85% and methicillin resistance at 22.22% in broiler chicken meat from selected slaughterhouses in Casablanca and Rabat areas, Morocco. This discovery underscores a potential public health concern. It is imperative to implement stringent preparation practices and hygiene measures throughout the food chain to mitigate the risk of MRSA transmission to consumers. Further investigations are also required, including molecular analysis of avian strains, comparing the genetic profile of avian strains with human clinical isolates.

## Figures and Tables

**Table 1:** Primer sequences of nuc, mecA, see, and sea genes.

**Gene**	**Primers**	**GenBank accession N°**	**Reference**
nuc	F: GCGATTGATGGTGATACGGTT	JX499023-6	[20]
	R: AGCCAAGCCTTGACGAACTAAAGC		
mecA	F: AAAATCGATGGTAAAGGTTGGC	Y00688	[21]
	R: AGTTCTGCAGTACCGGATTTGC		
sea	F: GGTTATCAATGTGCGGGTGG	M18970	[22]
	R: CGGCACTTTTTTCTCTTCGG		
see	F: AGGTTTTTTCACAGGTCATCC	M21319	[22]
	R: CTTTTTTTTCTTCGGTCAATC		

**Table 2:** Cycling mode of all tested genes according to the melting temperature of their primers [23].

**Step**	**Incubation**		**Cycles**
	**Temperature (°C)**	**Time**	
UDG activation	50	2 min	Hold
Dual-lock DNA polymerase	95	2 min	Hold
Denature	95	15 s	40
Anneal	55–60	15 s	
Extend	72	1 min	

**Table 3:** Presence of *Staphylococcus aureus* with corresponding Cq values in broiler chicken meat.

**Samples**	**Cq value**
1	26.99
2	28.67
3	28.41
4	26.63
5	25.43
6	18.53
7	26.33
8	26
9	26.77
10	28.31
11	27.72
12	27.8
13	27.65
14	29.04
15	32.62
16	30.19
17	26.92
18	27.51
19	27.2
20	26.31
21	22.68
22	28.26
23	27.81
24	26.39
25	27.04
26	27.09
27	28.41
28	33.81
29	26.8
30	15.79
31	30.01
32	28.4
33	27.28
34	23.89
35	33.53
36	27.62
37	27.26
38	29.1
39	26.18
40	26.5
41	27.75
42	28.23
43	28.25
44	14.8
45	27.89
46	28.48
47	31.23
48	28.84
49	27.56
50	28.19
51	27.08
52	31.77
53	30.81
54	15.66

*Note:* No Cq and Cq > 35 were considered negative.

Abbreviation: Cq, quantification cycle.

**Table 4:** Presence of the staphylococcal enterotoxin E (see) with corresponding Cq values in broiler chicken meat.

**Samples**	**Cq value**
1	No Cq
2	No Cq
3	34.55
4	No Cq
5	No Cq
6	No Cq
7	No Cq
8	No Cq
9	No Cq
10	No Cq
11	No Cq
12	No Cq
13	No Cq
14	No Cq
15	No Cq
16	No Cq
17	No Cq
18	No Cq
19	No Cq
20	No Cq
21	No Cq
22	No Cq
23	No Cq
24	No Cq
25	No Cq
26	No Cq
27	No Cq
28	No Cq
29	No Cq
30	No Cq
31	No Cq
32	No Cq
33	No Cq
34	No Cq
35	No Cq
36	No Cq
37	No Cq
38	No Cq
39	No Cq
40	No Cq
41	No Cq
42	No Cq
43	No Cq
44	No Cq
45	No Cq
46	No Cq
47	No Cq
48	No Cq
49	No Cq
50	No Cq
51	No Cq
52	No Cq
53	No Cq
54	No Cq

*Note:* No Cq and Cq > 35 were considered negative.

Abbreviation: Cq, quantification cycle.

**Table 5:** MRSA prevalence from broiler chicken meat with corresponding Cq values in broiler meat.

**Samples**	**Cq value**
1	No Cq
2	No Cq
3	No Cq
4	37.97
5	34.95
6	No Cq
7	No Cq
8	35.19
9	No Cq
10	36.58
11	34.73
12	33.94
13	34.68
14	34.94
15	No Cq
16	37.81
17	No Cq
18	32.59
19	35.15
20	No Cq
21	34.42
22	36.79
23	35.28
24	35
25	No Cq
26	33.93
27	35.18
28	No Cq
29	No Cq
30	39.77
31	No Cq
32	35.24
33	37.86
34	No Cq
35	No Cq
36	37.07
37	No Cq
38	39.42
39	No Cq
40	No Cq
41	No Cq
42	No Cq
43	34.71
44	36.04
45	No Cq
46	35.99
47	33.99
48	No Cq
49	No Cq
50	No Cq
51	35.05
52	No Cq
53	33.18
54	33.64

*Note:* No Cq and Cq > 35 were considered negative.

Abbreviation: Cq, quantification cycle.

## Data Availability

The authors declare that they have all the necessary data and are available where appropriate or requested by the editor.
